# Advances in Asymmetric Wettable Janus Materials for Oil–Water Separation

**DOI:** 10.3390/molecules27217470

**Published:** 2022-11-02

**Authors:** Jingjing Zhang, Congcong Wang, Huwei Xing, Qian Fu, Chenxi Niu, Lingbin Lu

**Affiliations:** Special Glass Key Lab of Hainan Province (Hainan University) & State Key Laboratory of Marine Resource Utilization in South China Sea, School of Materials Science and Engineering, Hainan University, Haikou 570228, China

**Keywords:** Janus materials, asymmetric wettability, oil–water separation, smart responsive, environmentally friendly

## Abstract

The frequent occurrence of crude oil spills and the indiscriminate discharge of oily wastewater have caused serious environmental pollution. The existing separation methods have some defects and are not suitable for complex oil–water emulsions. Therefore, the efficient separation of complex oil–water emulsions has been of great interest to researchers. Asymmetric wettable Janus materials, which can efficiently separate complex oil–water emulsions, have attracted widespread attention. This comprehensive review systematically summarizes the research progress of asymmetric wettable Janus materials for oil–water separation in the last decade, and introduces, in detail, the preparation methods of them. Specifically, the latest research results of two-dimensional Janus materials, three-dimensional Janus materials, smart responsive Janus materials, and environmentally friendly Janus materials for oil–water separation are elaborated. Finally, ongoing challenges and outlook for the future research of asymmetric wettable Janus materials are presented.

## 1. Introduction

With the progress of human civilization and the rapid development of the social industrial level, the problems of crude oil spills and random discharge of oily wastewater have become increasingly serious. A crude oil spill not only results in serious energy losses, but also pollutes the water environment, and causes irreversible damage to aquatic organisms, thus destroying the entire aquatic ecosystem [1,2,3,4]. Furthermore, some oils contain a variety of carcinogen substances, which can be concentrated in aquatic organisms, and then threaten human health through the food chain [5,6,7]. Therefore, the removal of oil from water and the separation of surfactant-containing oil–water emulsions have been the focus of attention [8,9,10]. Traditional oil–water separation methods including gravity, centrifugation, membrane separation, flotation, oxidation, etc., all have certain defects, such as low separation efficiency, high energy consumption, and secondary pollution [11,12,13,14]. Therefore, in order to solve the oil–water pollution problem, the development of new oil–water separation materials with high separation efficiency, low energy consumption, good stability, and environmental friendliness has become a challenge faced by researchers.

Oil in oily wastewater can be divided into three categories based on the diameter of the oil droplet. (1) Free oil: oil droplet diameter greater than 150 μm; (2) dispersed oil: oil droplet diameter less than 150 μm, greater than 20 μm; (3) emulsified oil: oil droplet diameter less than 20 μm. Among those, emulsions-dispersed micro/nano-droplets are the most difficult to separate. The usual oil–water separation methods are ineffective on them [15,16,17]. Emulsions are divided into water-in-oil emulsions and oil-in-water emulsions. In water-in-oil emulsions, oil is a continuous phase, and the droplet does not need to be broken for separation. In oil-in-water emulsions, oil is a dispersed phase, and dispersed in water in the form of small droplet, which needs to be broken for separating the oil from water [18]. Traditional demulsification methods include the thermochemical demulsification method, electrochemical demulsification method, etc. [19]. However, these traditional demulsification methods are usually complex and inefficient, prone to secondary contamination, and unsuitable for long-term operation.

**Figure 1:**
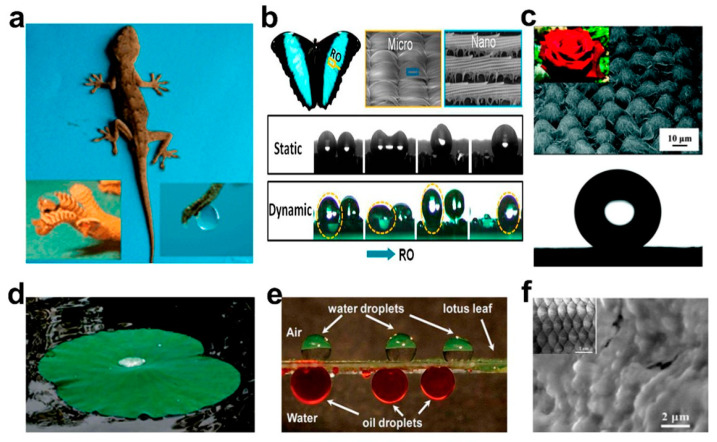
Special wettable surface in nature: (**a**) Photographs of a gecko under anesthesia. (**b**) Photo and SEM images of a Morpho deidamia butterfly, and fog droplet transportation on a static and dynamicbutterfly wing. (**c**) SEM image and digital photograph of a rose petal, and the spherical shape waterdrop on the surface. (**d**) Photograph of the upper surface of lotus leaf. (**e**) Water droplets on the upper surface of the lotus leaf and oil droplets on its lower surface underwater. (**f**) SEM image and optical image of fish scales. Adapted with permission from Ref. [37]. Copyright 2021, American Chemical Society.

It is known that a porous material can be used to separate oil–water mixtures when its surface has opposite wettability for water and oil [20,21]. Researchers have observed super-hydrophobic phenomena on the surfaces of many plants and animals in nature [22,23], such as lotus flowers [24], lotus leaves [25], geckos [26], and insects [27], as shown in Figure 1. After rigorous scientific argumentation, it was concluded that the special wettability of a material surface depended on two key factors: surface roughness and surface chemical composition [28,29,30]. Later, by mimicking the microstructure of these organisms with special wettability on surfaces, researchers developed some new special wettable porous materials with super-hydrophilic/oleophobic or super-hydrophobic/oleophilic properties, and then successfully applied them to oil–water separation. However, these single wettable materials can only remove oil or water unilaterally, which limits the application to complex oil–water emulsions [31]. Hence, single wettable materials still have limitations for oil–water separation.

**Figure 2:**
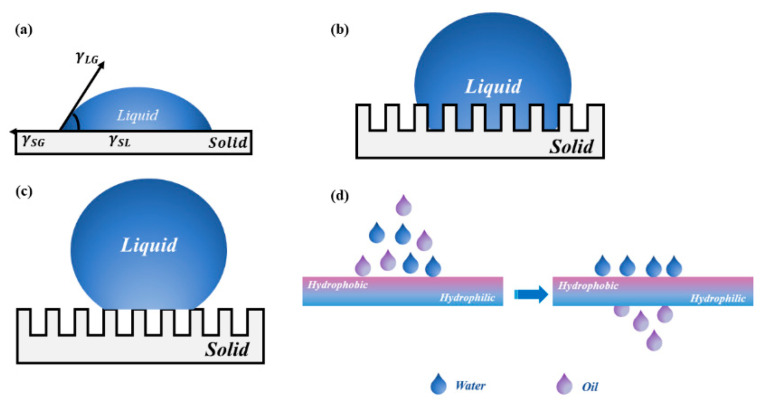
The liquid transmission mechanism: (**a**) Young’s model; (**b**) Wenzel model; (**c**) Cassie–Baxter model; (**d**) schematic diagram of liquid transmission of asymmetric wettable Janus material.

To solve the problem that the single wettable materials cannot effectively separate complex oil–water emulsions, researchers developed the asymmetric wettable Janus materials based on special wettable porous materials. The word “Janus” is derived from the ancient Greek God with two faces. It is similar to “yin-yang” in Tai Ji, which is part of Chinese traditional culture. It means the two opposite principles in nature. In 1985, Cho et al. [32] prepared structurally and compositionally asymmetrically polystyrene/polymethyl methacrylate Janus particles via seed emulsion polymerization, as the first report about Janus materials in the field of materials science. Subsequently, Gennes [33] used “Janus” to describe particles with different compositions or properties on both sides for the first time in his Nobel lecture in 1991. Since then, Janus materials have received widespread attention and have been explored intensively by researchers. In general, Janus materials can be divided into those with asymmetric wettability and those with asymmetric surface charges [34]. As the name implies, asymmetric wettable Janus materials are those materials with opposite wettable properties on each side, such as super-hydrophobic/super-hydrophilic [35,36] or super-oleophilic/super-oleophobic. The asymmetric wettability of porous materials can easily realize the directional transport of liquids and separation of various oil–water emulsions. The separation performance of oil–water separation materials depends on the wettability of their surface. The contact angle is the most intuitive numerical response to the wettability of the material surface. There are three main models for the contact angle of liquid on the surface of solid material: the Young’s model, the Wenzel model, and the Cassie–Baxter model. The liquid transmission mechanism and brief schematic diagram of the liquid transmission of asymmetric wettable Janus materials are shown in Figure 2. Asymmetric wettable Janus materials for oil–water separation can be divided into two-dimensional Janus materials and three-dimensional Janus materials according to their structural appearance.

**Figure 3:**
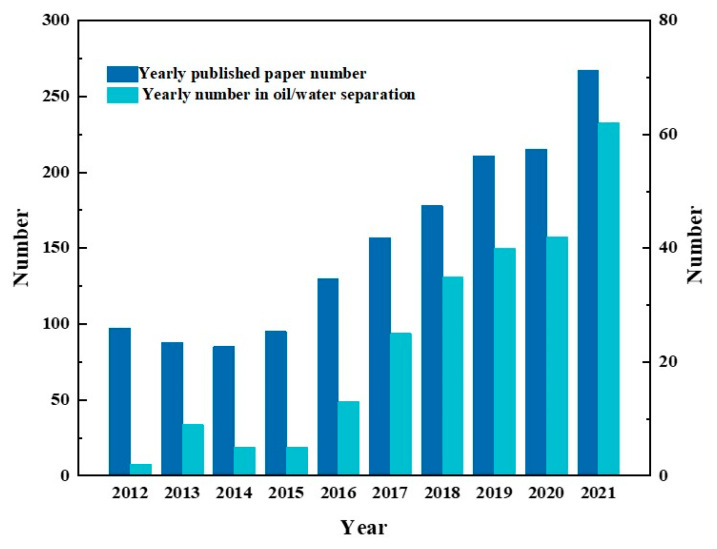
The date comparation of published articles on Janus materials and their application in oil/water separation retrieved from the Web of Science in the past decade using keywords ”Janus materials” and “oil/water separation’ or “oil-water separation”, respectively.

The statistics on the published articles about Janus materials in the past decade illustrate that the research interest on Janus materials for oil–water separation has a rising trend, as well as the number of articles shown in Figure 3. As a kind of new frontier material, asymmetric wettable Janus materials still have technical shortcomings and unknown potential awaiting further exploration and refinement. This paper reviews in detail the research progress of asymmetric wettable Janus materials for oil–water separation in the last decade and provides new research ideas for future research.

## 2. Preparation of Asymmetric Wettable Janus Materials

### 2.1. Layer-by-Layer Assembly Method

The layer-by-layer assembly method is the most straightforward method for constructing asymmetric wettable Janus materials [38,39,40,41]. In the layer-by-layer assembly method, two materials with opposite wettability are prepared separately and then assembled into an integrated unit physically or chemically. The concrete process includes materials compounding, electrostatic spinning, phase separation, etc. It has the advantages of controllable thickness and wettability, and relatively simple operation. Direct lamination of two materials with opposite wettability is the simplest and most straightforward method in the layer-by-layer assembly method.

**Figure 4:**
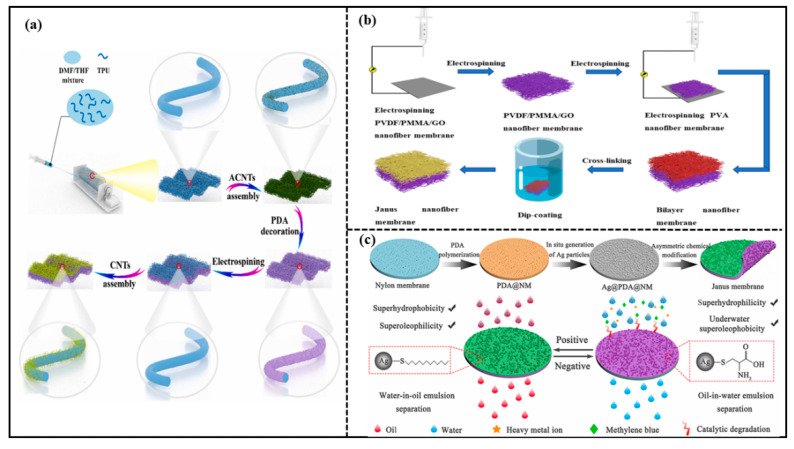
(**a**) Schematic demonstration of the overall preparation procedure of Janus membrane (JM). Reprint with permission from Ref. [42]. Copyright 2022, Elsevier. (**b**) schematic demonstration of Janus membrane preparation process. Reprint with permission from Ref. [43]. Copyright 2022, MDPI.; (**c**) schematic illustration for the fabrication and applications of Janus membrane. Reprint with permission from Ref. [48]. Copyright 2022, Elsevier.

Electrostatic spinning technology is an effective method commonly used to prepare membranes with micro-diameter or nano-diameter fibers and highly porous structures. A nanofiber structure prepared via electrostatic spinning technology has unique properties such as high specific surface area and high porosity. Hence, the electrostatic spinning technology is introduced to prepare the Janus membranes. For example, Huang et al. [42] proposed a simple “carbon nanotube (CNT) decoration and nanofiber membrane integration” method to produce a mechanically strong Janus membrane (JM). The schematic demonstration of the overall preparation procedure of the JM is shown in Figure 4a. The JM consisted of a super-hydrophilic nanofiber composite layer and a hydrophobic nanofiber composite membrane with the thickness of the hydrophobic layer controlled by the electrostatic spinning. The JM enabled the on-demand separation of various oily wastewaters, including oil–water mixtures with different oil densities, oil-in-water emulsions, and water-in-oil emulsions. The JM presented high separation fluxes and separation efficiencies. Wu et al. [43] also prepared a Janus membrane with excellent oil–water separation capacity via the electrostatic spinning technique. A mixture of polyvinylidene fluoride (PVDF), polymethyl methacrylate (PMMA), and graphene oxide (GO) was firstly electrostatically spun to form a hydrophobic layer, then polyvinyl alcohol (PVA) nanofibers were coated on the hydrophobic membrane by the layer-by-layer electrostatic spinning to form a composite membrane. Finally, the composite membrane was cross-linked to obtain the Janus membrane. The schematic demonstration of the overall preparation procedure of JM is shown in Figure 4b. The separation efficiency of the Janus membrane remained almost unchanged after several oil–water separation experiments with an oil–water separation flux of 1909.9 L·m^−2^·h^−1^. The separation efficiency reached 99.9%.

As a well-established technology, the electrostatic spinning is a preferred method used by researchers to prepare Janus membranes. However, electrostatic spinning can be affected by several factors during operation, and has high demands on the environment, raw materials, and parameter settings. Generally, Janus materials prepared by the layer-by-layer assembly method have a clear boundary between two layers, which are not easily compatible with each other. Therefore, Janus materials prepared by the layer-by-layer assembly method tend to separate between two layers and lead to less stability and less durability.

### 2.2. Single-Sided Modification Method

Another kind of method for preparing Janus materials is the single-sided modification method [44,45,46]. The single-sided modification is to modify only one side of a material, endowing a material with opposite wettability on both sides. This method is popular among researchers because of its greater flexibility and variety of approaches. Common approaches include single-sided spraying, chemical deposition, and in situ growth, etc.

Hu et al. [47] prepared a Janus hollow fiber membrane (JHFM) by co-depositing polydopamine and polyelectrolyte on the outer surface of the polypropylene hollow fiber microfiltration membrane to form a thin electrophilic layer with controlled thickness. JHFM continuously could separate surfactant-stabilized oil-in-water emulsions with efficiency up to 99%. Zheng et al. [48] successfully prepared a Janus nylon membrane with super-hydrophilic/super-oleophobic properties on the front side and super-hydrophobic/submerged super-oleophobic properties on the back side as shown in Figure 4c. In this work, asymmetric chemical modification was conducted with dodecyl mercaptan and L-cysteine on the positive and negative surfaces, respectively. The Janus nylon membrane separated oil-in-water and water-in-oil emulsions on both sides with efficiencies over 99%.

Researchers have successfully developed several asymmetric wettable Janus materials based on different substrates with the single-sided modification method. Some excellent results have been achieved in the field of oil–water separation. Various preparation methods of Janus materials with asymmetric wettability for oil-water separation are summarized in Table 1. However, the single-sided modification method still has some disadvantages that cannot be ignored. During single-sided spray modification, the liquid modifier is inevitably drawn into the interior of porous material due to the capillary effect. If the distance of spraying and the amount of modifier are not accurately controlled, then the modifier will penetrate into the interior of the substrate. The result will make the modified surface an uncontrollable thickness and even the whole substrate involved, and finally cause the desired single-sided modification failure. Therefore, more research needs be performed to overcome the disadvantages of the single-sided modification and find more convenient modification approaches.

## 3. Two-Dimensional Asymmetric Wettable Janus Materials

Two-dimensional (2D) materials are usually referred to as membrane materials, which separate oil–water emulsions based on the filtration principle. Before asymmetric wettable Janus membranes were developed, hydrophobic/oleophilic membranes were used to separate water-in-oil emulsions, and hydrophilic/oleophobic membranes were used to separate oil-in-water emulsions. As the emulsions separation process relies on the sieving effect of porous membranes based on pore size, water or oil droplets collect and adsorb on the membrane surface. At the same time, smaller droplets can also clog the porous structure of membranes slowly. It will cause membrane fouling problems and thus affect the oil–water separation effect [49,50]. As an emerging membrane material, asymmetric wettable Janus membranes can separate water-in-oil and oil-in-water emulsions by filtration. The synergistic effect of asymmetric wettability can prevent the interaction between emulsion and the membrane surface, reduce contamination of the membrane surface, and provide some anti-fouling effect, thus ensuring the separation performance of the membrane [51,52,53,54,55,56].

**Figure 5:**
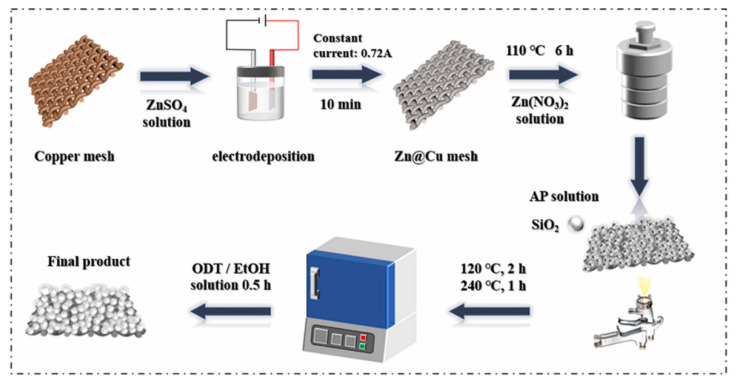
Schematic illustration for the fabrication process of the Janus membrane. Reprint with permission from Ref. [59]. Copyright 2022, Elsevier.

Janus membrane materials are the earliest developed and most researched class of Janus materials. Various 2D Janus materials with asymmetric wettability for oil-water separation are summarized in Table 2. Pan et al. [57] prepared an asymmetric wettable Janus membrane for oil–water separation with the buoyancy of polyvinylidene fluoride nanofibers at the water/air interface. ZnO nanowires grown on the surface of nanofibers in situ mimicked the nanoscale dogwood structure. The Janus membrane showed good hydrophilicity and oleophobicity at the side modified by ZnO nanowires, and the opposite hydrophobicity on the other side. It was allowed to be used as a “water removal” or “oil removal” filter. Driven by gravity alone, the membrane had excellent permeability with fluxes of 1210 L·m^−2^·h^−1^ and 7653 L·m^−2^·h^−1^ for water and oil, respectively. This membrane demonstrated excellent durability with the separation efficiency above 97.24% after ten cycles. Zuo et al. [58] prepared an asymmetric wettable Janus polyvinylidene fluoride (PVDF) membrane by thermally induced phase separation (TIPS) and spraying methods. The preparation was carried out by the rapid deposition of polydopamine/polyethyleneimine (PDA/PEI) and micro/nano SiO_2_ particles cross-linked with dichloro diphenyl silane on two surfaces of the PVDF membrane, respectively. The prepared Janus PVDF membrane showed excellent separation performance for both oil-in-water and water-in-oil emulsions. Xu et al. [59] obtained a Janus membrane with asymmetric wettability by growing super-hydrophilic ZnO on copper grids via a simple hydrothermal method and then modifying the copper grids via spraying SiO_2_ and octadecanethiol. The schematic illustration for the fabrication process of the Janus membrane is shown in Figure 5. The separation efficiency of the Janus membrane was over 99% for both light and heavy oil mixtures. It could maintain good performance even if after acid alkali corrosion, wear, and circulation experiments. Hence, the Janus membrane showed a great potential in the fields of oil–water separation and environmental protection.

In addition to the initial pursuit of separating oil–water emulsions, researchers have also investigated the antimicrobial properties of Janus materials for oil–water separation and endowed antimicrobial properties to some oil–water separation materials. Zhang et al. [60] selected cellulose paper as the substrate and prepared the Janus cellulose nanocomposite membrane with super-hydrophobic and super-oleophilic properties. PVDF/hydrophobic SiO_2_ nanocomposites were assembled onto the top of the substrate and adhering to PU via both high-pressure spraying and electrostatic spinning techniques. This membrane could separate water-in-oil and oil-in-water emulsions and had the advantages of wear resistance, and acid and alkali resistance at the same time. More importantly, it could be easily degraded and did not burden the ecological environment after discarded. Lv et al. [61] successfully prepared an antimicrobial Janus cellulose membrane via immobilizing Ag nanoparticles on a fibrous membrane. One side loaded with Ag nanoparticles of the membrane was firstly protected with tape, then the other side was modified to hydrophobicity via impregnation. The Janus cellulose membrane provided effective gravity-driven separation for oil-in-water and water-in-oil emulsions, and it was kept from bacterial contamination during oil–water separation at the same time, which could avoid the loss to a certain extent.

The 2D asymmetric wettable Janus materials have the advantages of high permeate fluxes and good separation results. However, Janus membranes often require specific instruments or strict conditions during preparation. These requirements present an obstacle to the industrial production. Moreover, Janus membranes still suffer from poor stability and limited stain resistance, which limit the long-term use of Janus membranes. Therefore, the improvement and innovation of preparation for 2D Janus membranes will be a common challenge for researchers in the future.

## 4. Three-Dimensional Asymmetric Wettable Janus Materials

Three-dimensional (3D) asymmetric wettable Janus materials separate oil–water emulsions based on both adsorption and filtration principles. Compared to 2D Janus materials, 3D Janus materials are more versatile as they can not only separate oil–water emulsions, but also temporarily store the separated water or oil. The emergence of 3D Janus materials solves the problems of low porosity, and inappropriate pore size of 2D Janus materials. Moreover, 3D Janus materials have the advantages of low density, high specific surface area, and easy modification, which make it particularly suitable for oil–water separation.

**Figure 6:**
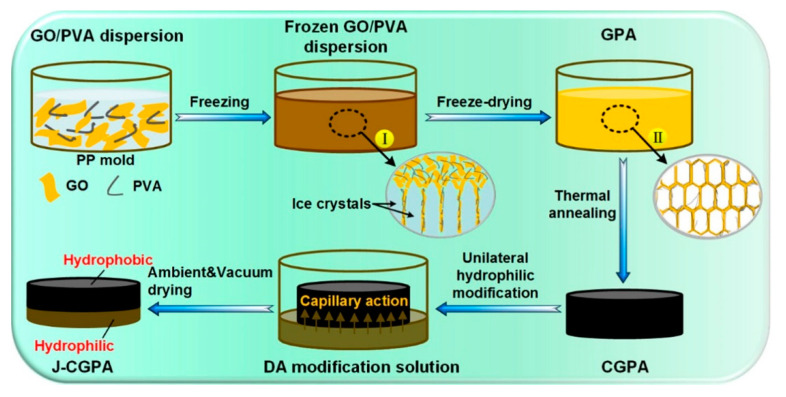
Schematic illustration of the preparation process of J-CGPA aerogel. Reprint with permission from Ref. [66]. Copyright 2019, American Chemical Society.

**Figure 7:**
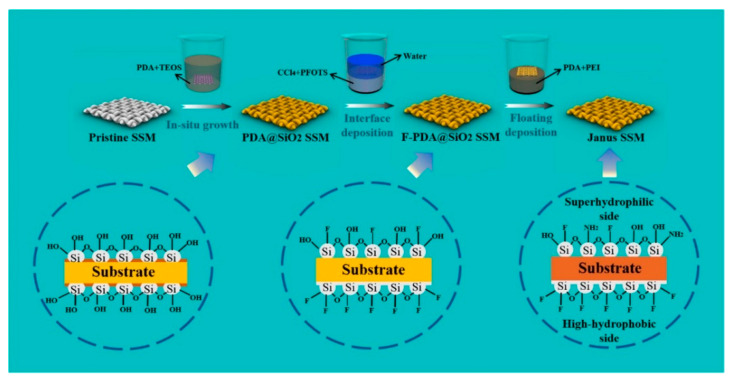
Schematic illustration of the preparation process of Janus SSM. Reprint with permission from Ref. [68]. Copyright 2022, Elsevier.

The preparation of 3D Janus materials is generally conducted via preparing a porous block material firstly, and then modifying the porous block material unilaterally. Li et al. [66] developed a high-quality graphene/polyvinyl alcohol Janus aerogel (J-CGPA) via preparing aerogel with a simple direct freeze-forming technique firstly, and then it was modified by hydrophilicity on one side. The preparation process is shown in Figure 6. J-CGPA showed completely opposite wettability on the two sides. One side was super-hydrophobic (WCA, 143°), while the other side was super-hydrophilic. J-CGPA showed remarkable separation properties for highly emulsified oil-in-water and water-in-oil emulsions, as well as stratified oil–water mixtures. The material had excellent separation efficiency and good feasibility in practical application. Thangavelu et al. [67] designed a two-step method to prepare an amphiphilic Janus cellulose acetate-molybdenum disulfide (CA-MoS_2_) fiber sponge. The CA-MoS_2_ fiber sponge had an excellent separation property even after five cycles. Liu et al. [68] prepared Janus stainless steel meshes (SSM) with super-hydrophilic and super-hydrophobic properties on each side. The preparation process is shown in Figure 7. The Janus SSM could separate light/heavy oil from oil–water mixtures efficiently. Based on these results, a 3D-Janus-SSM bed (PDA@ SiO_2_SSM) was designed via assembling a single Janus SSM and 20 super-hydrophilic SSMs grown in situ. The 3D-Janus-SSM bed had long and irregular permeation channels that allowed droplets to collide, coalesce, and break up during the separation process. It exhibited good emulsion separation capabilities too. For oil-in-water emulsions, the separation efficiency was up to 99.31% with a flux greater than 2864 L·m^−2^·h^−1^. For water-in-oil emulsions, the separation efficiency was greater than 99.68% with a flux of 7072 L·m^−2^·h^−1^. Liu et al. [69] provided a facile and environmentally friendly method to prepare a Janus copper foam via the vapor phase deposition and water droplet-controlled infiltration technology. The Janus copper foam was driven by gravity and the interfacial affinity between oil and water. It had high separation efficiency and excellent repeatability. This strategy is an important reference for the design of 3D Janus materials. Actually, most preparation processes of 3D Janus materials are costly, non-green, and complex; therefore, researchers are looking for simple, green, and efficient ways.

The 3D Janus materials reported so far have shown high permeation flux and separation efficiency for oil–water separation. The separation process can be completed by the liquid’s gravity alone without any external force. All these promise a bright future of 3D Janus materials. However, due to the relatively short period of research, there are plenty of unsolved issues about 3D Janus materials. The 3D Janus materials consist of two layers with a certain thickness. However, the bonding strength between the two layers of 3D Janus materials prepared by the layer-by-layer assembly method may not be strong enough. Additionally, the thickness of two layers of 3D Janus materials prepared by the single-sided modification method is hard to control precisely. All these have adverse effects on the stability and durability of 3D Janus materials. Compared to 2D Janus materials, 3D Janus materials are more difficult to prepare. Future research on 3D Janus materials should focus on these issues.

## 5. Smart Responsive Asymmetric Wettable Janus Materials

Smart responsive asymmetric wettable Janus materials are a kind of novel material whose surface wettability can happen to change under certain stimuli. As a smart oil–water separation material, it allows the selective penetration of water or oil in response to specific stimuli from an external environment, that is, the manipulable oil–water separation performance. Smart responsive Janus materials are generally prepared by coating or grafting stimuli-responsive polymers onto the surface of matrix. According to the external stimulus types, they are classified as pH-responsive, thermal-responsive, light-responsive, and electric-responsive Janus materials.

### 5.1. pH-Responsive Asymmetric Wettable Janus Materials

The pH-responsive Janus materials have the advantages of rapid response and ease of operation. In order to achieve a shift in the wettability under different pH conditions, it is necessary to add ionized groups or functional groups to the surface of substrates. Usually, ionized groups and functional groups, which can undergo protonation and deprotonation under different pH conditions, are carboxyl (-COOH), amino (-NH_2_), amide bonds, etc. [70,71]. Currently, pH-responsive polymers have been applied to porous substrates such as metallic meshes, fibrous membranes, filter papers, and sponge to achieve manipulable oil–water separation [72,73]. 

**Figure 8:**
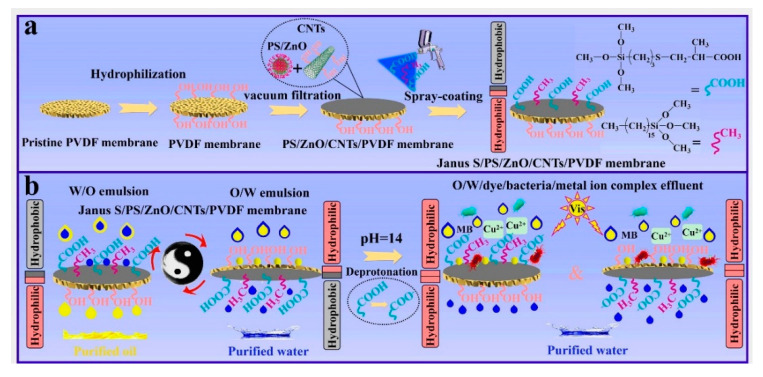
(**a**) Schematic for the preparation process of the Janus S/PS/ZnO/CNTs/PVDF composite membrane and (**b**) its use for membrane separation of surfactant-stabilized W/O and O/W emulsions and O/W/dye/bacteria/metal-ion-complex wastewater. Reprint with permission from Ref. [76]. Copyright 2022, Elsevier.

In 2008, Zhang et al. [74] firstly reported a novel pH-responsive surface with switchable wettability at different pH conditions. The material exhibited super-hydrophilicity at low pH and super-hydrophobicity at high pH. Subsequently, Wen et al. [75] proposed a method to prepare a pH-responsive fabric. After polymerizing dopamine in situ on the fabric and immersing in a cysteamine solution, a sulfhydrylation fabric was obtained. Next, hydrophobic stearyl methacrylate and undecylenic acid with carboxyl groups (-COOH) were introduced to the fabric surface by the photoinduced coupling reaction between sulfhydryl groups and olefins. Due to the protonation and deprotonation effects of carboxyl groups, this fabric achieved a rapid conversion between super-hydrophilicity and super-hydrophobicity in solutions with different pH. It was used for the separation of various oil–water mixtures with a separation efficiency of 99%. Hu et al. [76] synthesized a pH-responsive monomer via a classical thiol-alkene reaction. Then, they successfully prepared a pH-responsive Janus membrane by immobilizing the monomer on PVDF membrane. The schematic illustration for the fabrication process of the Janus membrane is shown in Figure 8. This Janus membrane could efficiently separate a wide range of complex oil–water emulsions. More importantly, the pH-responsive Janus membrane successfully treated complex wastewaters containing oil, bacteria, organic dyes, and metal ions. Qu et al. [70] also designed a smart multifunctional pH-responsive fabric with reversible wettability. The fabric could switch repeatedly between super-hydrophobicity/super-oleophilicity and super-hydrophilicity/submerged super-oleophobicity with recyclability. Due to this ingenious response mechanism, the resulting fabric could continuously separate various complex oil–water mixtures in situ with high separation efficiency and flux. In addition, the fabric maintained excellent durability and chemical stability under harsh environments. Some encouraging progress has been made in pH-responsive asymmetric wettable Janus materials. Further improvement in the pH sensitivity of the material needs constant efforts. Furthermore, various polymers were introduced in pH-responsive Janus materials. The environmental toxicity of these polymers must be taken into account to avoid secondary contamination.

### 5.2. Thermal-Responsive Asymmetric Wettable Janus Materials

Among the smart responsive asymmetric wettable Janus materials, the separation process of thermal-responsive Janus materials is simpler to operate and responsive. The oil–water separation performs at a specific temperature so that temperature is the only key factor, which need be adjusted in a specific range to achieve a shift in wettable properties. Thermal-responsive materials are generally prepared via grafting some thermosensitive compounds on the surface. The surface and structure of the grafted material will change with the change of temperature. Thus, a change in surface wettability is achieved. Currently, thermal-responsive polymers are usually used for constructing a temperature responsive function.

Poly (N-isopropyl acrylamide) (PNIPAM) is the best-known thermal-responsive polymer by far [77], which has been present in many available thermal-responsive Janus materials for oil–water separation. Wei et al. [78] prepared an antifouling Janus membrane with a temperature-sensitive bead-like skin for oil–water separation via electrostatic spray and electrostatic spinning. This membrane could successfully separate water-in-oil and oil-in-water emulsions in the range of 20–50 °C. PNIPAM expanded and stretched below 20 °C, while it condensed above 50 °C. The PNIPAN transformation is reversible so that the reusability of this material is guaranteed. However, thermosensitive materials contain polymer components and require strict temperature control. Hence, the poor heat resistance of polymer limits the available temperature range inevitably.

### 5.3. Light-Responsive Asymmetric Wettable Janus Materials

It has been shown that some transition metal oxides (e.g., TiO_2_, ZnO, etc.), which possess excellent photocatalytic and photosensitivity, can achieve surface wettability conversion under visible or UV light irradiation [79,80,81,82]. Light-responsive polymeric materials with functional groups, such as spiro-pyran, diarylethylene, or azobenzene, undergo rapid photoisomerization when exposed to specific wavelengths of light. Hence, they can be used to construct switchable interfaces for bidirectional oil–water separation.

Researchers have used such transition metal oxides to prepare light-responsive asymmetric wettable materials for oil–water separation. Ma et al. [83] immobilized modified TiO_2_ nanoparticles on the fabric surface and then modified the fabric to a super-hydrophobic surface with perfluorooctanoic acid as a hydrophobic modifier. The obtained fabric could convert its surface wettability to super-hydrophilic under UV light irradiation, while turning back to the initial super-hydrophobicity after heating. In addition, many organic compounds possess the properties of reversible conformational transformation under light stimulation, which can also be used to prepare light responsive materials. For example, the N=N bond of azo compounds undergoes bond breakage and rotation under UV light irradiation. The breakage and rotation cause cis-trans isomerization of the structure and realize wettability shift. Kollarigowda et al. [84] prepared a membrane whose surface could instantaneously shift from the hydrophobic to hydrophilic state under the irradiation of UV light. The hydrophobic fluorocarbon silane was linked with azobenzene by a covalent bond and then grafted onto the surface of the cellulose membrane by PDA. The obtained cellulose membrane can be used for oil–water separation as well as water purification. The cellulose membrane could also adsorb various organic reagents with good reusability. Although the wettability transition on the surface of light-responsive Janus materials can be achieved rapidly, the change in wettability is slight in many cases. The reversible wettability transition between two extreme wettable states is still difficult to achieve. Hence, it is a major obstacle to practical application.

### 5.4. Electric-Responsive Asymmetric Wettable Janus Materials

**Figure 9:**
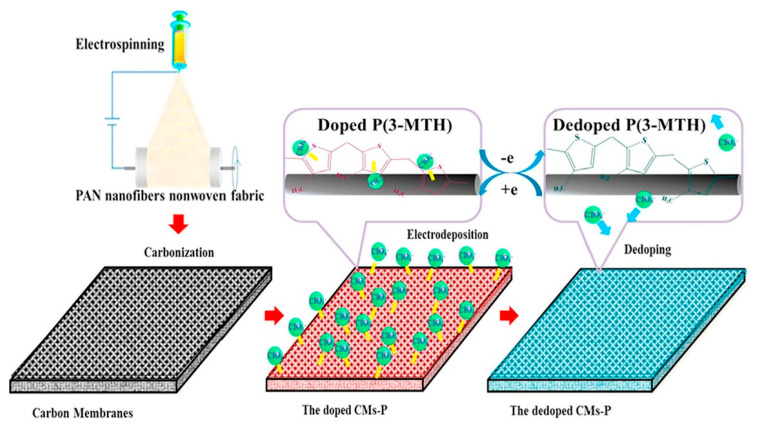
The schematic of CMs-P fabrication process. Reprint with permission from Ref. [81]. Copyright 2020, Elsevier.

Compared with other types of smart responsive asymmetric wettable Janus materials, electric-responsive Janus materials can finish the wettability conversion in a short time and are considered to be a more effective and feasible smart responsive controllable oil–water separation material. The surface wettability of electric-responsive Janus materials is changed by controlling voltage, which not only reduces the surface tension between the solid and liquid interface, but also triggers a redox reaction on the surface to change the surface chemistry composition. Thus, the surface wettability happens to shift. Du et al. [85] designed an electric-responsive poly-3-methyl thiophene/carbon nanofiber membrane with the switchable conversion of super-hydrophobic and super-hydrophilic wettability. An electric carbon nanofiber membrane with good electrical conductivity was selected as the substrate, functionalized with 3-methyl thiophene. The membrane achieved reversible doping and de-doping of ClO^4−^ on the surface at the corresponding oxidation or reduction potentials. The schematic of the CMs-P fabrication process is shown in Figure 9. The controlled transition properties between hydrophilicity and hydrophobicity made it qualified to separate oil–water mixtures and emulsions. This work provided a new insight into the design of controllable membranes. Various smart responsive Janus materials with asymmetric wettability for oil-water separation are summarized in Table 3.

## 6. Environmentally Friendly Asymmetric Wettable Janus Materials

With the increasing attention on environmental protection, the research on asymmetric wettable Janus materials for oil–water separation has developed toward environmental-friendly, green, and sustainable orientation. Harmful raw materials are used as little as possible while maintaining the oil–water separation properties of materials. Biodegradable materials, such as cellulose and polylactic acid (PLA), are selected as new raw materials to develop oil–water separation materials. Some great achievements have been made.

**Figure 10:**
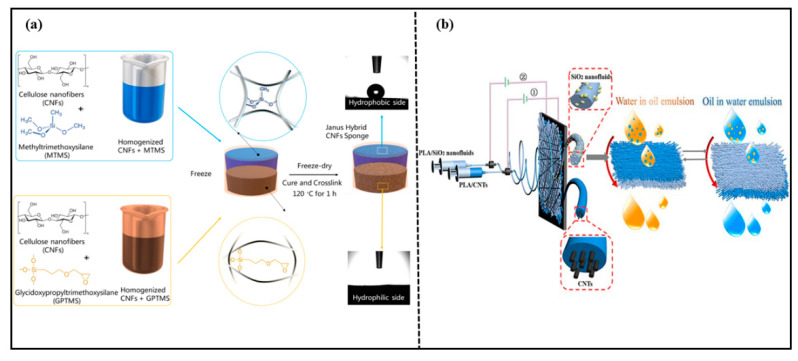
(**a**) Preparation of Janus hybrid sustainable all-CNF sponge. Reprint with permission from Ref. [87]. Copyright 2021, Elsevier.; (**b**) preparation route for Janus PLA hybrid fibrous membranes and the oil/water separation process. Reprint with permission from Ref. [63]. Copyright 2020, American Chemical Society.

Cellulose is the most abundant natural polymer on earth [86]. It is biocompatible, biodegradable, thermally stable, and chemically stable. Therefore, degradable cellulose-based Janus materials for oil–water separation have become a popular research topic. Yu et al. [64] prepared a novel cellulose acetate (CA) fiber membrane via the plasma gas phase grafting technology and polymerization of octamethylcyclotetrasiloxane on CA. The CA fiber membrane could separate water-in-oil emulsions efficiently. Wang et al. [65] prepared a biomimetic Janus wood membrane inspired by the oriented tubular voids and graded scaffolds of wood. The membrane was prepared via removing a part of hemicellulose and lignin from natural balsa wood. Then, an octadecanethiol/ethanol solution was sprayed on one side of the delignified wood. The Janus wood membrane exhibited asymmetric wettability after UV irradiation. The Janus wood membrane had unidirectional water transfer capability and selective oil–water separation. Agaba et al. [73,87] reported a Janus all-cellulose nanofiber sponge for efficient oil–water separation as shown in Figure 10a. The Janus sponge with asymmetric wettability was obtained via free-drying two suspensions with different wettability. The sponge had high elasticity, high porosity, and efficient separation properties for oil–water emulsions driven by gravity alone, as well as anti-fouling properties. Fei et al. [88] designed a three-dimensional Janus structure based on the hydrophobic matrix and constructed a 3D Janus structural cellulose aerogel with asymmetric wettability via an in situ construction strategy from hydrophobicity to asymmetric wettability. The 3D Janus structure cellulose aerogel exhibited opposite wettability on two sides. Due to the asymmetric wettability and interconnected network structure, the material achieved an efficient separation performance for various oil–water mixtures, even water-in-oil emulsions, with a permeate flux up to 3121 L·m^−2^·h^−1^ and separation efficiency of 99.5%. This study provided a new construction strategy and a scientifically significant reference for environmentally friendly Janus porous materials. Various 3D Janus materials with asymmetric wettability for oil-water separation are summarized in Table 4.

PLA is a biodegradable material made from starch, which is extracted from renewable plant resources. It can be degraded completely by microorganisms in nature after being discarded. Qin et al. [63] prepared a Janus-PLA fibrous membrane with a PLA/carbon nanotube (CNTs) fibrous membrane spun in static electricity, following a PLA/SiO_2_ nanofluid (NFS) membrane spun in static electricity onto one side of the PLA/CNTs’ fibrous membrane. The preparation route for the Janus-PLA hybrid fibrous membrane and the oil/water separation process are shown in Figure 10b. The strong electrostatic interactions between SiO_2_-NFS and carbon nanotubes made the prepared Janus-PLA fiber membrane superior stability. The Janus-PLA fiber membrane could separate oil–water emulsions efficiently with high separation efficiency even after eleven cycles.

The number of studies on environmentally friendly Janus materials for oil–water separation is still small at present. Most of the reported environmentally friendly Janus materials selected cellulose as the raw material. Although the use of cellulose as a raw material implements the sustainable concept, the durability of the resulting material is relatively poor. Environmentally friendly Janus materials still have great prospects for development in the future.

## 7. Summary and Outlook

This paper reviews the latest research progress and results of asymmetric wettable Janus materials for oil–water separation in the past decade. It can be seen that Janus materials have received more and more attention in recent years. The research of Janus materials has been broadened and deepened. The types of Janus materials have gradually expanded from the initial 2D Janus materials to 3D Janus materials and smart responsive Janus materials. They have excellent oil–water separation capabilities, especially for the efficient separation of surfactant-stabilized oil–water emulsions. However, the research of asymmetric wettable Janus materials still faces some challenges.

(1) Compared to 2D Janus materials, 3D Janus materials have a more practical application value and broader development prospects. More research on 3D Janus materials are appealed. Degradable raw materials should be selected in the preparation process, such as photodegradable materials and biodegradable materials. Convenient preparation methods need be developed to reduce preparation difficulty, which could facilitate industrial mass production and practical application. What’s more, it is necessary for oil–water separation materials to be recycled.
(2) The smart responsive Janus materials possess better intelligent performance for on-demand oil–water separation. The existing research is focused on 2D smart responsive materials. The 3D smart responsive materials are more advanced than 2D smart responsive materials. Hence, it is suggested that researchers can introduce smart responsive monomers to develop 3D smart responsive materials in the future.
(3) Researchers in various fields are now moving in the direction of environmental friendliness, sustainability, and greenness. It is the future trend of asymmetric wettable Janus materials too. Although the current asymmetric wettable Janus materials have been available to perform oil–water separation excellently, most of them could lead to secondary contamination during the preparation process and after being discarded. The causes of secondary contamination include non-degradable raw materials, toxic and harmful reagents in the preparation process, and non-recyclable materials. Therefore, the future development of asymmetric wettable Janus materials should focus on finding environmentally friendly, renewable, and degradable resources as raw materials; optimizing the preparation process; and avoiding the use of toxic and harmful reagents. It is expected that environmentally friendly Janus materials will be paid more attention in the future.

## Figures and Tables

**Table 1:** Various preparation methods of Janus materials with asymmetric wettability for oil–water separation.

Material	Method	Refs.
Janus membrane	Electrospinning	[42]
Janus membrane	Electrospinning	[43]
Co-doped graphene aerogel	Hydrothermal reduction combined with electrostatic spraying method	[44]
Janus carbon nanotube membrane	Plasma treatment	[45]
Janus membrane	Atomic layer deposition	[46]
Janus hollow fiber membrane	Co-depositing	[47]
Janus nylon membrane	In situ generation	[48]

**Table 2:** Summary of 2D Janus materials with asymmetric wettability for oil–water separation.

Material	Method	Emulsion	Separation Efficiency (Max)	Flux (L·m^−2^·h^−1^) (Max)	Recyclable	Refs.
CPVA-PVDF/PMMA/GO Janus membrane	Layer-by-layer electrospinning	O/W W/O	99.9%	1909.9	Yes	[43]
Janus cellulose nanocomposite membrane	High-pressure spraying and electrospinning	O/W W/O	>88%	2238	Yes	[60]
Janus nanofibrous membrane	Electrospinning	O/W W/O	99%	6652	Yes	[42]
Janus hollow fiber membrane	Co-deposited; assembled	O/W	>99%	/	Yes	[47]
Janus nylon membrane	In situ generation	O/W W/O	>99%	1700	Yes	[48]
Janus membrane	Co-deposited	O/W W/O	>97%	1526 ± 177	Yes	[62]
Janus PVDF membrane	Cold plasma modification	O/W W/O	>99.8%	9300	Yes	[50]
Scalable Janus membrane	Cyclic self-assembly; single-side spray coating	O/W W/O	>99.9%	23,800	Yes	[52]
Graphene oxide-connected cotton fibers membrane	Impregnation	Oil/water mixture	>99.8%	95,000	Yes	[53]
Janus cotton	In situ dual-phase formation; volatilization induced fabrication.	Oil/water mixture	98%	/	Yes	[54]
Janus polyvinylidene fluoride membrane	Non-solvent induced phase separation (NIPS)	O/W W/O	99.72%	489.6 ± 91.2	Yes	[55]
Janus nanofibrous membrane	Electrospinning	W/O Oil/water mixture	99.98%	/	Yes	[56]
Janus PLA membrane	Electrospinning	O/W W/O Oil/water mixture	>99%	1142–1185	Yes	[63]
Janus cellulose acetate fiber membrane	Plasma gas phase grafting	W/O	98.81%	331.72	Yes	[64]
Janus delignified wood membrane	Single-sided spray coating	Oil/water mixture	>99.3%	2450	Yes	[65]
2D nanoneedle-like ZnO/SiO_2_ Janus membrane	Single-sided spray coating	Oil/water mixture	>99.99%	13,067.5	Yes	[59]

**Table 3:** Summary of smart responsive Janus materials with asymmetric wettability for oil–water separation.

Material	Response Mode	Method	Emulsion	Separation Efficiency (Max)	Flux (L·m^−2^·h^−1^) (Max)	Recyclable	Refs.
Functionalized fabric with pH-sensitivity	pH-response	In situ growth	Oil/water mixture	99.5%	9362	Yes	[70]
Smart Janus membrane	pH-response	Single-side spray coating	O/W W/O	99%	4917	Yes	[76]
Thermo-modulated nanofibrous skin covered Janus membrane	Thermal-response	Synergetic electro-spraying/electrospinning	O/W W/O	99%	6000	Yes	[78]
Porous ZnO@copper membrane	Light-response	Atomic layer deposition; low-temperature hydrothermal	O/W oil/water mixture	>99.7%	40,000	Yes	[79]
Robust super-hydrophobic mesh coated by PANI/TiO_2_ nanoclusters	Light-response	Nanoclusters coating	Oil/water mixture	99.7%	176,000	Yes	[81]
Electro-responsive carbon membrane	Electric-response	Coating	O/W W/O oil/water mixture	>99.5%	/	Yes	[85]

**Table 4:** Summary of 3D Janus materials with asymmetric wettability for oil–water separation.

Material	Method	Emulsion	Separation Efficiency (Max)	Flux (L·m^−2^·h^−1^) (Max)	Recyclable	Refs.
Janus graphene oxide sponge	Freeze drying	O/W W/O	≥99.2%	/	Yes	[89]
Graphene/poly (vinyl alcohol) Janus aerogel	Floating-coating	O/W W/O	99.7%	1306	Yes	[66]
3D Janus mesh bed	In situ growth	O/W W/O	>99.68%	>7072	Yes	[68]
Janus copper foam	Alkaline erosion	Oil/water mixture	93%	/	Yes	[69]
Janus hybrid sustainable all-cellulose nanofiber sponge	Freeze drying	Oil/water mixture	100%	3300	Yes	[87]
Cellulose acetate-MoS_2_ amphiphilic Janus-like fibrous sponge	Hydrothermal method; drop-casting	O/W	99%	/	Yes	[67]
Three-dimensional Janus cellulose aerogel	Vacuum-assisted chemical vapor deposition; coating	W/O oil/water mixture	99%	3121	Yes	[88]
Scalable Janus membrane	Cyclic self-assembly; single-side spray coating	O/W W/O	>99.9%	23,800	Yes	[52]

## Data Availability

The data presented in this study are available on request from the corresponding author.

## References

[1] Chen J., Zhang W., Wan Z., Li S., Huang T., Fei Y. (2019). Oil Spills from Global Tankers: Status Review and Future Governance. J. Clean. Prod..

[2] Bengani-Lutz P., Zaf R.D., Culfaz-Emecen P.Z., Asatekin A. (2017). Extremely Fouling Resistant Zwitterionic Copolymer Membranes with ~ 1 Nm Pore Size for Treating Municipal, Oily and Textile Wastewater Streams. J. Membr. Sci..

[3] Zeng X., Qian L., Yuan X., Zhou C., Li Z., Cheng J., Xu S., Wang S., Pi P., Wen X. (2017). Inspired by *Stenocara* Beetles: From Water Collection to High-Efficiency Water-in-Oil Emulsion Separation. ACS Nano.

[4] Tran N.H., Reinhard M., Gin K.Y.-H. (2018). Occurrence and Fate of Emerging Contaminants in Municipal Wastewater Treatment Plants from Different Geographical Regions-a Review. Water Res..

[5] Almojjly A., Johnson D., Oatley-Radcliffe D.L., Hilal N. (2018). Removal of Oil from Oil-Water Emulsion by Hybrid Coagulation/Sand Filter as Pre-Treatment. J. Water Process Eng..

[6] Cheng Y., Li X., Xu Q., Garcia-Pineda O., Andersen O.B., Pichel W.G. (2011). SAR Observation and Model Tracking of an Oil Spill Event in Coastal Waters. Mar. Pollut. Bull..

[7] Gupta R.K., Dunderdale G.J., England M.W., Hozumi A. (2017). Oil/Water Separation Techniques: A Review of Recent Progresses and Future Directions. J. Mater. Chem. A.

[8] Abdulredha M.M. (2022). Water-in-Oil Emulsion Stability and Demulsification via Surface-Active Compounds: A Review. J. Pet. Sci. Eng..

[9] Ismail N.H., Salleh W.N.W., Ismail A.F., Hasbullah H., Yusof N., Aziz F., Jaafar J. (2020). Hydrophilic Polymer-Based Membrane for Oily Wastewater Treatment: A Review. Sep. Purif. Technol..

[10] Kücük Ş., Hejase C.A., Kolesnyk I.S., Chew J.W., Tarabara V.V. (2021). Microfiltration of Saline Crude Oil Emulsions: Effects of Dispersant and Salinity. J. Hazard. Mater..

[11] Deng Y., Zhang G., Bai R., Shen S., Zhou X., Wyman I. (2019). Fabrication of Superhydrophilic and Underwater Superoleophobic Membranes via an in Situ Crosslinking Blend Strategy for Highly Efficient Oil/Water Emulsion Separation. J. Membr. Sci..

[12] Zhu Y., Wang D., Jiang L., Jin J. (2014). Recent Progress in Developing Advanced Membranes for Emulsified Oil/Water Separation. NPG Asia Mater..

[13] Song J., Huang S., Lu Y., Bu X., Mates J.E., Ghosh A., Ganguly R., Carmalt C.J., Parkin I.P., Xu W. (2014). Self-Driven One-Step Oil Removal from Oil Spill on Water via Selective-Wettability Steel Mesh. ACS Appl. Mater. Interfaces.

[14] Chew N.G.P., Zhang Y., Goh K., Ho J.S., Xu R., Wang R. (2019). Hierarchically Structured Janus Membrane Surfaces for Enhanced Membrane Distillation Performance. ACS Appl. Mater. Interfaces.

[15] Tanudjaja H.J., Hejase C.A., Tarabara V.V., Fane A.G., Chew J.W. (2019). Membrane-Based Separation for Oily Wastewater: A Practical Perspective. Water Res..

[16] Tummons E.N., Tarabara V.V., Chew J.W., Fane A.G. (2016). Behavior of Oil Droplets at the Membrane Surface during Crossflow Microfiltration of Oil–Water Emulsions. J. Membr. Sci..

[17] Zhang W., Liu N., Cao Y., Lin X., Liu Y., Feng L. (2017). Superwetting Porous Materials for Wastewater Treatment: From Immiscible Oil/Water Mixture to Emulsion Separation. Adv. Mater. Interfaces.

[18] Zhu X., Dudchenko A., Gu X., Jassby D. (2017). Surfactant-Stabilized Oil Separation from Water Using Ultrafiltration and Nanofiltration. J. Membr. Sci..

[19] Bhushan B. (2019). Bioinspired Oil–Water Separation Approaches for Oil Spill Clean-up and Water Purification. Philos. Trans. R. Soc. Math. Phys. Eng. Sci..

[20] Qu M., Liu Q., He J., Li J., Liu L., Yang C., Yang X., Peng L., Li K. (2020). A Multifunctional Superwettable Material with Excellent PH-Responsive for Controllable in Situ Separation Multiphase Oil/Water Mixture and Efficient Separation Organics System. Appl. Surf. Sci..

[21] Tummons E., Han Q., Tanudjaja H.J., Hejase C.A., Chew J.W., Tarabara V.V. (2020). Membrane Fouling by Emulsified Oil: A Review. Sep. Purif. Technol..

[22] Wang H., Zhou H., Niu H., Zhang J., Du Y., Lin T. (2015). Dual-Layer Superamphiphobic/Superhydrophobic-Oleophilic Nanofibrous Membranes with Unidirectional Oil-Transport Ability and Strengthened Oil-Water Separation Performance. Adv. Mater. Interfaces.

[23] Liang Y., Kim S., Kallem P., Choi H. (2019). Capillary Effect in Janus Electrospun Nanofiber Membrane for Oil/Water Emulsion Separation. Chemosphere.

[24] Yong J., Chen F., Yang Q., Huo J., Hou X. (2017). Superoleophobic Surfaces. Chem. Soc. Rev..

[25] Wang Z., Yang J., Song S., Liu X., Li S. (2021). A Bio-Inspired Method to Fabricate the Substrate-Independent Janus Membranes with Outstanding Floatability for Precise Oil/Water Separation. Bull. Mater. Sci..

[26] Zhang R., Sun Y., Guo Z., Liu W. (2021). Janus Membranes with Asymmetric Wettability Applied in Oil/Water Emulsion Separations. Adv. Sustain. Syst..

[27] Gao X., Jiang L. (2004). Water-Repellent Legs of Water Striders. Nature.

[28] Li J.-J., Zhou Y.-N., Luo Z.-H. (2017). Mussel-Inspired V-Shaped Copolymer Coating for Intelligent Oil/Water Separation. Chem. Eng. J..

[29] Du X., You S., Wang X., Wang Q., Lu J. (2017). Switchable and Simultaneous Oil/Water Separation Induced by Prewetting with a Superamphiphilic Self-Cleaning Mesh. Chem. Eng. J..

[30] Li J., Kang R., Tang X., She H., Yang Y., Zha F. (2016). Superhydrophobic Meshes That Can Repel Hot Water and Strong Corrosive Liquids Used for Efficient Gravity-Driven Oil/Water Separation. Nanoscale.

[31] Zhou C., Cheng J., Hou K., Zhao A., Pi P., Wen X., Xu S. (2016). Superhydrophilic and Underwater Superoleophobic Titania Nanowires Surface for Oil Repellency and Oil/Water Separation. Chem. Eng. J..

[32] Cho I., Lee K.-W. (1985). Morphology of Latex Particles Formed by Poly(Methyl Methacrylate)-Seeded Emulsion Polymerization of Styrene. J. Appl. Polym. Sci..

[33] de Gennes P.-G. (1992). Soft Matter (Nobel Lecture). Angew. Chem. Int. Ed. Engl..

[34] Yang H., Xie Y., Hou J., Cheetham A.K., Chen V., Darling S.B. (2018). Janus Membranes: Creating Asymmetry for Energy Efficiency. Adv. Mater..

[35] Yang X., Wang Z., Shao L. (2018). Construction of Oil-Unidirectional Membrane for Integrated Oil Collection with Lossless Transportation and Oil-in-Water Emulsion Purification. J. Membr. Sci..

[36] Wang H., Ding J., Dai L., Wang X., Lin T. (2010). Directional Water-Transfer through Fabrics Induced by Asymmetric Wettability. J. Mater. Chem..

[37] Zheng W., Huang J., Li S., Ge M., Teng L., Chen Z., Lai Y. (2021). Advanced Materials with Special Wettability toward Intelligent Oily Wastewater Remediation. ACS Appl. Mater. Interfaces.

[38] Yang H., Wei F., Hu K., Lyu J. (2017). Effects of Mud Slurry on Flow Resistance of Cohesionless Coarse Particles. Powder Technol..

[39] Ranganath A.S., Baji A. (2018). Electrospun Janus Membrane for Efficient and Switchable Oil–Water Separation. Macromol. Mater. Eng..

[40] An Y.-H., Yu S.J., Kim I.S., Kim S.-H., Moon J.-M., Kim S.L., Choi Y.H., Choi J.S., Im S.G., Lee K.E. (2017). Hydrogel Functionalized Janus Membrane for Skin Regeneration. Adv. Healthc. Mater..

[41] Wang Y., Zhang L., Wu J., Hedhili M.N., Wang P. (2015). A Facile Strategy for the Fabrication of a Bioinspired Hydrophilic–Superhydrophobic Patterned Surface for Highly Efficient Fog-Harvesting. J. Mater. Chem. A.

[42] Huang X., Wu Z., Zhang S., Xiao W., Zhang L., Wang L., Xue H., Gao J. (2022). Mechanically Robust Janus Nanofibrous Membrane with Asymmetric Wettability for High Efficiency Emulsion Separation. J. Hazard. Mater..

[43] Wu H., Shi J., Ning X., Long Y.-Z., Zheng J. (2022). The High Flux of Superhydrophilic-Superhydrophobic Janus Membrane of CPVA-PVDF/PMMA/GO by Layer-by-Layer Electrospinning for High Efficiency Oil-Water Separation. Polymers.

[44] Ren X., Guo H., Ma X., Hou G., Chen L., Xu X., Chen Q., Feng J., Si P., Zhang L. (2018). Improved Interfacial Floatability of Superhydrophobic and Compressive S, N Co-Doped Graphene Aerogel by Electrostatic Spraying for Highly Efficient Organic Pollutants Recovery from Water. Appl. Surf. Sci..

[45] Abraham S., Ma G., Montemagno C.D. (2016). Janus Carbon Nanotube Membranes by Selective Surface Plasmoxidation. Adv. Mater. Interfaces.

[46] Waldman R.Z., Yang H., Mandia D.J., Nealey P.F., Elam J.W., Darling S.B. (2018). Janus Membranes via Diffusion-Controlled Atomic Layer Deposition. Adv. Mater. Interfaces.

[47] Hu Y.-Q., Li H.-N., Xu Z.-K. (2022). Janus Hollow Fiber Membranes with Functionalized Outer Surfaces for Continuous Demulsification and Separation of Oil-in-Water Emulsions. J. Membr. Sci..

[48] Zheng L., Li H., Lai X., Huang W., Lin Z., Zeng X. (2022). Superwettable Janus Nylon Membrane for Multifunctional Emulsion Separation. J. Membr. Sci..

[49] Rezaei M., Warsinger D.M., Lienhard V.J.H., Duke M.C., Matsuura T., Samhaber W.M. (2018). Wetting Phenomena in Membrane Distillation: Mechanisms, Reversal, and Prevention. Water Res..

[50] Lin Y., Salem M.S., Zhang L., Shen Q., El-shazly A.H., Nady N., Matsuyama H. (2020). Development of Janus Membrane with Controllable Asymmetric Wettability for Highly-Efficient Oil/Water Emulsions Separation. J. Membr. Sci..

[51] Kota A.K., Kwon G., Choi W., Mabry J.M., Tuteja A. (2012). Hygro-Responsive Membranes for Effective Oil–Water Separation. Nat. Commun..

[52] Fu C., Gu L., Zeng Z., Xue Q. (2020). Simply Adjusting the Unidirectional Liquid Transport of Scalable Janus Membranes toward Moisture-Wicking Fabric, Rapid Demulsification, and Fast Oil/Water Separation. ACS Appl. Mater. Interfaces.

[53] Yang S., Li J., Yang N., Sha S., Yang C., Zhao J., Duoerkun A., Hong Y., Wu C. (2021). Underwater Superoleophobic Graphene Oxide-Connected Cotton Fibers Membrane for Antifouling Oil/Water Separation. J. Water Process Eng..

[54] Liang L., Li X., Hu H., Zhang J., Peng Y., Liu C., Yang M. (2021). Preparation of a Novel Janus-Cotton via the in-Situ Dual-Phase Formation and Volatilization-Induced Fabrication Method for Oil/Water Separation with Single and Double Layers. J. Environ. Chem. Eng..

[55] Yang H., Wang Y., Fang S., Wang G., Zhu L., Zeng Z., Wang L. (2021). Janus Polyvinylidene Fluoride Membranes with Controllable Asymmetric Configurations and Opposing Surface Wettability Fabricated via Nanocasting for Emulsion Separation. Colloids Surf. Physicochem. Eng. Asp..

[56] Cheng C., Wei Z., Gu J., Wu Z., Zhao Y. (2022). Rational Design of Janus Nanofibrous Membranes with Novel Under-Oil Superhydrophilic/Superhydrophobic Asymmetric Wettability for Water-in-Diesel Emulsion Separation. J. Colloid Interface Sci..

[57] Pan T., Li Z., Shou D., Shou W., Fan J., Liu X., Liu Y. (2019). Buoyancy Assisted Janus Membrane Preparation by ZnO Interfacial Deposition for Water Pollution Treatment and Self-cleaning. Adv. Mater. Interfaces.

[58] Zuo J.-H., Gu Y.-H., Wei C., Yan X., Chen Y., Lang W.-Z. (2020). Janus Polyvinylidene Fluoride Membranes Fabricated with Thermally Induced Phase Separation and Spray-Coating Technique for the Separations of Both W/O and O/W Emulsions. J. Membr. Sci..

[59] Xu Y., Zeng X., Qiu L., Yang F. (2022). 2D Nanoneedle-like ZnO/SiO2 Janus Membrane with Asymmetric Wettability for Highly Efficient Separation of Various Oil/Water Mixtures. Colloids Surf. Physicochem. Eng. Asp..

[60] Zhang M., Yang Q., Gao M., Zhou N., Shi J., Jiang W. (2021). Fabrication of Janus Cellulose Nanocomposite Membrane for Various Water/Oil Separation and Selective One-Way Transmission. J. Environ. Chem. Eng..

[61] Lv Y., Li Q., Hou Y., Wang B., Zhang T. (2019). Facile Preparation of an Asymmetric Wettability Janus Cellulose Membrane for Switchable Emulsions’ Separation and Antibacterial Property. ACS Sustain. Chem. Eng..

[62] Ma H.-Y., Hu Y.-N., Yang H., Zhu L.-J., Wang G., Zeng Z.-X., Wang L.-H. (2021). In Situ Mussel-Inspired Janus Membranes Using Catechol and Polyethyleneimine as the Additives for Highly Efficient Oil/Water Emulsions Separation. Sep. Purif. Technol..

[63] Qin Y., Shen H., Han L., Zhu Z., Pan F., Yang S., Yin X. (2020). Mechanically Robust Janus Poly(Lactic Acid) Hybrid Fibrous Membranes toward Highly Efficient Switchable Separation of Surfactant-Stabilized Oil/Water Emulsions. ACS Appl. Mater. Interfaces.

[64] Yu X., Zhang X., Xing Y., Zhang H., Jiang W., Zhou K., Li Y. (2021). Development of Janus Cellulose Acetate Fiber (CA) Membranes for Highly Efficient Oil–Water Separation. Materials.

[65] Wang K., Liu X., Dong Y., Zhang S., Li J. (2022). A Biomimetic Janus Delignified Wood Membrane with Asymmetric Wettability Prepared by Thiol-Ol Chemistry for Unidirectional Water Transport and Selective Oil/Water Separation. Colloids Surf. Physicochem. Eng. Asp..

[66] Li Y., Zhang G., Gao A., Cui J., Zhao S., Yan Y. (2019). Robust Graphene/Poly(Vinyl Alcohol) Janus Aerogels with a Hierarchical Architecture for Highly Efficient Switchable Separation of Oil/Water Emulsions. ACS Appl. Mater. Interfaces.

[67] Thangavelu K., Ravaux F., Zou L. (2021). Cellulose Acetate-MoS 2 Amphiphilic Janus-like Fibrous Sponge for Removing Oil from Wastewater. Environ. Technol. Innov..

[68] Liu Z., Zuo J., Zhao T., Chen Z., Zeng X., Chen M., Xu S., Cheng J., Wen X., Pi P. (2022). A 3D Janus Stainless Steel Mesh Bed with High Efficiency and Flux for On-Demand Oil-in-Water and Water-in-Oil Emulsion Separation. Sep. Purif. Technol..

[69] Liu C., Peng Y., Huang C., Ning Y., Shang J., Li Y. (2022). Bioinspired Superhydrophobic/Superhydrophilic Janus Copper Foam for On-Demand Oil/Water Separation. ACS Appl. Mater. Interfaces.

[70] Qu M., Liu Q., Liu L., Yang C., Yuan S., Shi F., Peng L., Xiong S., He J. (2021). A Superwettable Functionalized-Fabric with PH-Sensitivity for Controlled Oil/Water, Organic Solvents Separation, and Selective Oil Collection from Water-Rich System. Sep. Purif. Technol..

[71] Jin L., Wang Y., Xue T., Xie J., Xu Y., Yao Y., Li X. (2019). Smart Amphiphilic Random Copolymer-Coated Sponge with PH-Switchable Wettability for On-Demand Oil/Water Separation. Langmuir.

[72] Dang Z., Liu L., Li Y., Xiang Y., Guo G. (2016). In Situ and Ex Situ PH-Responsive Coatings with Switchable Wettability for Controllable Oil/Water Separation. ACS Appl. Mater. Interfaces.

[73] Cheng Z., Wang J., Lai H., Du Y., Hou R., Li C., Zhang N., Sun K. (2015). PH-Controllable On-Demand Oil/Water Separation on the Switchable Superhydrophobic/Superhydrophilic and Underwater Low-Adhesive Superoleophobic Copper Mesh Film. Langmuir.

[74] Zhang Q., Xia F., Sun T., Song W., Zhao T., Liu M., Jiang L. (2008). Wettability Switching between High Hydrophilicity at Low PH and High Hydrophobicity at High PH on Surface Based on PH-Responsive Polymerw. Chem. Commun..

[75] Zeng X., Yang K., Huang C., Yang K., Xu S., Wang L., Pi P., Wen X. (2019). Novel PH-Responsive Smart Fabric: From Switchable Wettability to Controllable On-Demand Oil/Water Separation. ACS Sustain. Chem. Eng..

[76] Hu J., Gui L., Zhu M., Liu K., Chen Y., Wang X., Lin J. (2022). Smart Janus Membrane for On-Demand Separation of Oil, Bacteria, Dye, and Metal Ions from Complex Wastewater. Chem. Eng. Sci..

[77] Kim Y.-J. (2017). Thermo-Responsive Polymers and Their Application as Smart Biomaterials. J. Mater. Chem. B.

[78] Wei B., Wang K., Wang J., Zhang T.C., Tian X., Yuan S., Li Y., Chang H., Liang Y., Zhou Z. (2022). Thermo-Modulated Nanofibrous Skin Covered Janus Membranes for Efficient Oil/Water Separation. Colloids Surf. Physicochem. Eng. Asp..

[79] Huang A., Kan C.-C., Lo S.-C., Chen L.-H., Su D.-Y., Soesanto J.F., Hsu C.-C., Tsai F.-Y., Tung K.-L. (2019). Nanoarchitectured Design of Porous ZnO@copper Membranes Enabled by Atomic-Layer-Deposition for Oil/Water Separation. J. Membr. Sci..

[80] Duan Z., Zhao Z., Luo D., Zhao M., Zhao G. (2016). A Facial Approach Combining Photosensitive Solgel with Self-Assembly Method to Fabricate Superhydrophobic TiO_2_ Films with Patterned Surface Structure. Appl. Surf. Sci..

[81] Wang J., Wang X., Zhao S., Sun B., Wang Z., Wang J. (2020). Robust Superhydrophobic Mesh Coated by PANI/TiO_2_ Nanoclusters for Oil/Water Separation with High Flux, Self-Cleaning, Photodegradation and Anti-Corrosion. Sep. Purif. Technol..

[82] He Y., Wan M., Wang Z., Zhang X., Zhao Y., Sun L. (2019). Fabrication and Characterization of Degradable and Durable Fluoride-Free Super-Hydrophobic Cotton Fabrics for Oil/Water Separation. Surf. Coat. Technol..

[83] Ma L., He J., Wang J., Zhou Y., Zhao Y., Li Y., Liu X., Peng L., Qu M. (2019). Functionalized Superwettable Fabric with Switchable Wettability for Efficient Oily Wastewater Purification, in Situ Chemical Reaction System Separation, and Photocatalysis Degradation. ACS Appl. Mater. Interfaces.

[84] Kollarigowda R.H., Bhyrappa H.M., Cheng G. (2019). Stimulus-Responsive Biopolymeric Surface: Molecular Switches for Oil/Water Separation. ACS Appl. Bio Mater..

[85] Du L., Quan X., Fan X., Chen S., Yu H. (2019). Electro-Responsive Carbon Membranes with Reversible Superhydrophobicity/Superhydrophilicity Switch for Efficient Oil/Water Separation. Sep. Purif. Technol..

[86] Moon R.J., Martini A., Nairn J., Simonsen J., Youngblood J. (2011). Cellulose Nanomaterials Review: Structure, Properties and Nanocomposites. Chem. Soc. Rev..

[87] Agaba A., Marriam I., Tebyetekerwa M., Yuanhao W. (2021). Janus Hybrid Sustainable All-Cellulose Nanofiber Sponge for Oil-Water Separation. Int. J. Biol. Macromol..

[88] Fei Y., Tan Y., Deng Y., Xia P., Cheng J., Wang C., Zhang J., Niu C., Fu Q., Lu L. (2022). *In Situ* Construction Strategy for Three-Dimensional Janus Cellulose Aerogel with Highly Efficient Oil–Water Separation Performance: From Hydrophobicity to Asymmetric Wettability. Green Chem..

[89] Yun J., Khan F.A., Baik S. (2017). Janus Graphene Oxide Sponges for High-Purity Fast Separation of Both Water-in-Oil and Oil-in-Water Emulsions. ACS Appl. Mater. Interfaces.

